# Induction of Short-Term Sensitization by an Aversive Chemical Stimulus in Zebrafish Larvae

**DOI:** 10.1523/ENEURO.0336-19.2020

**Published:** 2020-12-03

**Authors:** Adam C. Roberts, Joseph B. Alzagatiti, Duy T. Ly, Julia M. Chornak, Yuqi Ma, Asif Razee, Gohar Zavradyan, Umair Khan, Julia Lewis, Aishwarya Natarajan, Alisher Baibussinov, Jasmine Emtage, Meghna Komaranchath, Jared Richards, Michelle Hoang, Jason Alipio, Emma Laurent, Amit Kumar, C. S. Campbell, Rebecca Stark, Javier Carmona, Anjum Hussain, Courtney Scaramella, Jenan Husain, Reed Buck, Ava Jafarpour, Miguel Garcia, Steve Mendoza, Gerardo Sandoval, Brandon Agundez, Amanda Fink, Emily Deutsch, Sarah C. Hernandez, Katsushi Arisaka, David L. Glanzman

**Affiliations:** 1 Department of Psychology, California State University at Fullerton, Fullerton, CA 92831; 2 Department of Integrative Biology and Physiology, University of California, Los Angeles, Los Angeles, CA 90095; 3 Department of Neuroscience, University of California, Los Angeles, Los Angeles, CA 90095; 4 Department of Psychology, University of California, Los Angeles, Los Angeles, CA 90095; 5 Department of Molecular, Cell, and Developmental Biology, University of California, Los Angeles, Los Angeles, CA 90095; 6 Department of Microbiology, Immunology, and Molecular Genetics, University of California, Los Angeles, Los Angeles, CA 90095; 7 Department of Psychology, Rowan University, Glassboro, NJ 08028; 8 Department of Biology, Brown University, Providence, RI 02912; 9 Department of Psychology, California State University at San Bernardino, San Bernardino, CA 92407; 10 School of Biology, University of St. Andrews, St. Andrews, Fife, Scotland, UK; 11 Department of Physics, University of California, Los Angeles, Los Angeles, CA 90095; 12 Department of Neurobiology, David Geffen School of Medicine at UCLA, Los Angeles, CA 90095; 13 Integrative Center for Learning and Memory, Brain Research Institute, David Geffen School of Medicine at UCLA, Los Angeles, CA 90095

**Keywords:** behavioral plasticity, learning, memory, sensitization, zebrafish

## Abstract

Larval zebrafish possess a number of molecular and genetic advantages for rigorous biological analyses of learning and memory. These advantages have motivated the search for novel forms of memory in these animals that can be exploited for understanding the cellular and molecular bases of vertebrate memory formation and consolidation. Here, we report a new form of behavioral sensitization in zebrafish larvae that is elicited by an aversive chemical stimulus [allyl isothiocyanate (AITC)] and that persists for ≥30 min. This form of sensitization is expressed as enhanced locomotion and thigmotaxis, as well as elevated heart rate. To characterize the neural basis of this nonassociative memory, we used transgenic zebrafish expressing the fluorescent calcium indicator GCaMP6 (Chen et al., 2013); because of the transparency of larval zebrafish, we could optically monitor neural activity in the brain of intact transgenic zebrafish before and after the induction of sensitization. We found a distinct brain area, previously linked to locomotion, that exhibited persistently enhanced neural activity following washout of AITC; this enhanced neural activity correlated with the behavioral sensitization. These results establish a novel form of memory in larval zebrafish and begin to unravel the neural basis of this memory.

## Significance Statement

We have discovered a form of short-term behavioral sensitization in zebrafish larvae. Because the larvae are translucent, neural activity related to sensitization memory can be optically monitored in the intact and, in some cases behaving, fish using a genetically encoded ratiometric calcium indicator, GCaMP6. Taking advantage of this capability, we succeeded in identifying a region in the hindbrain that may mediate, at least in part, the memory for sensitization in the zebrafish larva. These findings initiate an understanding of how activity in this region mediates a simple form of nonassociative memory in a relatively simple vertebrate animal.

## Introduction

Sensitization, an enhanced behavioral response because of aversive or arousing stimuli, such as those resulting from a predatory attack, has been documented in a phylogenetically diverse set of organisms (Thompson and Spencer, 1966; Carew et al., 1971; Duerr and Quinn, 1982; Krasne and Glanzman, 1986; Rankin et al., 1990; Fendt et al., 1994; Koch, 1999; Watkins et al., 2010; Cai et al., 2012). Neurobiological investigations of sensitization memory have progressed most successfully in invertebrate organisms possessing relatively simple nervous systems (Davis, 2011; Byrne and Hawkins, 2015). A particularly important model system for cell biological analyses of sensitization has been the defensive reflex of the marine snail *Aplysia californica*; researchers exploiting this system have made significant progress toward understanding sensitization at the molecular, cellular, and systems levels (Glanzman et al., 1989, 1990; Sugita et al., 1992; Cleary and Byrne, 1993; Kaang et al., 1993; White et al., 1993; Xu et al., 1994; Hegde et al., 1997; Martin et al., 1997; Rajasethupathy et al., 2012; Byrne and Hawkins, 2015; Hu et al., 2015). The success of this invertebrate model argues that an understanding of the biological basis of sensitization in vertebrates could be more readily achieved by initially investigating this form of learning in a vertebrate with a less complex nervous system than that of mammals. Larval zebrafish appear particularly well suited for neurobiological investigations of simple forms of learning and memory. They possess only ∼100,000 neurons at 5 d postfertilization (dpf); while still large compared with the number of neurons in the central nervous systems of many invertebrates, this number is significantly less than that in the mammalian brain. In addition to the relative simplicity of their nervous systems, zebrafish larvae are highly amenable to genetic (Douglass et al., 2008; Arrenberg et al., 2009; Del Bene and Wyart, 2012; Portugues et al., 2013) and pharmacological manipulation (Goldsmith, 2004; Best et al., 2008; Roberts et al., 2011; Wolman et al., 2011). Furthermore, zebrafish larvae are translucent, a property that facilitates optical investigations of learning-related changes in neuronal structure and neuronal activity in the intact brain using genetically encoded fluorescent molecules, including calcium indicators (Sagasti et al., 2005; Meyer and Smith, 2006; Ahrens et al., 2013; Zada et al., 2014; Son et al., 2016). These advantages have made the larval zebrafish increasingly attractive to neurobiologists who wish to understand memory formation (Amsterdam et al., 1999; Goldsmith, 2004; Kotani et al., 2006; Scott et al., 2007; Asakawa and Kawakami, 2008; Baier and Scott, 2009; Rihel et al., 2010; Bedell et al., 2012; Dahlem et al., 2012; Moore et al., 2012; Hwang et al., 2013; Nelson et al., 2020).

However, the sophistication of the tools that can be harnessed to investigate memory formation in zebrafish larvae have far outpaced the discovery of memory-related behavioral changes amenable to experimental analysis in these animals (Roberts et al., 2013). This is partly because the technologies commonly used to investigate neural systems in zebrafish are most effective early in development, when the behavioral repertoire of these animals is relatively limited. To fully exploit the advantages of zebrafish larvae as a model biological system for understanding memory, it is critical to discover forms of memory that they can express at ∼5 dpf. Toward that end, we now report that zebrafish of this age are capable of behavioral sensitization. Specifically, we have found that several behaviors in zebrafish at 5–6 dpf can be sensitized by exposure to an aversive agent [allyl isothiocyanate (AITC)]. Although sensitization elicited by AITC relies on transient receptor potential (TRP) channels, it appears to be independent of inflammatory processes. The memory for this sensitization persists for up to 30 min. In addition, we have identified a specific neural correlate of this memory.

## Materials and Methods

### Animals

After collection, zebrafish eggs were put into E3 water (5 mm NaCl, 0.33 mm MgSO_4_, 0.33 mm CaCl_2_, 0.17 mm KCl, and 10^−5^% methylene blue; pH 7.2) and placed in an incubator (28.5°C). Zebrafish were maintained in E3 (rearing medium) through development and this was the medium used for most experimental procedures. In some experiments, 1 mm HEPES was added to the E3 medium for increased buffering. Behavioral experiments were performed on the TL strain of zebrafish obtained from the UCLA core facility, whereas the imaging experiments used transgenic fish expressing GCaMP6s pan neuronally, Tg(elav3:GCaMP6s) (RRID: ZFIN ID: ZDB-TGCONSTRCT-141023–2; Vladimirov et al., 2014).

### Behavioral protocols

#### Measurements of tail movements in semi-restrained fish

Larval zebrafish, 3–12 dpf (mixed sex), were embedded in 3% low melting point agarose and then positioned in a cell culture dish. After the agarose had solidified, the tail and a portion of the head were freed from the agarose to permit tail movements and to allow the surface of the head to be directly exposed to a chemical irritant (AITC), respectively. The culture dishes containing the fish were then placed on a light box (Gagne Inc.) to record tail movements with a high-speed digital camera (Exilim ExFH25: Casio America); the recording frame rate was 120 or 240 frames/s (fr/s). The fish were given 20 or 30 min to acclimate to the experimental arrangement before video recording. Zebrafish exhibit a variety of tail movements (Budick and O'Malley, 2000). Here, however, we did not differentiate among types of tail movements; rather, we simply measured the duration of an animal’s movement (swimming duration) and the number of times its tail moved in either direction from the midline (tail flicks). We measured tail movements in response to an ejection of bath solution (100 μl) toward the head or spontaneous tail movements before, after, or during exposure to AITC (30-s duration). In control experiments, the semi-restrained fish were exposed to E3 instead of AITC. The data were normalized by subtracting the pretest (baseline) values from the posttest values.

#### Measurements of swimming activity in freely moving fish

For experiments investigating motor activity in freely moving fish (5 dpf), animals were placed in small Petri dishes (36 mm in diameter) containing 14 ml of E3 medium and allowed to acclimate for 30 min. Subsequently, the level of activity to a pretest stimulus (ejection of 50 μl of E3 from a hand-held micropipette directed toward the fish’s head using moderate, albeit unquantified, force) was measured. This was done by recording the total distance the fish swam using a high-speed camera (240 fr/s) for a period of 30 or 60 s immediately after the ejection of E3. The position of the fish was assessed every 10 fr. Following the measurement of activity in response to the pretest stimulus, each fish was exposed for 30 s to either AITC or E3; the AITC/E3 was then rapidly washed out using ∼2 dish volumes of E3, after which the distance traveled by the fish (sampled every 10 fr) was subsequently measured for a 30/60-s period at specified times.

#### Measurements of thigmotaxis in freely moving fish

To determine whether a brief exposure to AITC causes a persistent increase in thigmotaxis, 20 fish were placed in a Petri dish (50 mm in diameter) containing a 12-ml volume of E3 and allowed to acclimate for 1 h. After this period of acclimation, the fish were exposed to AITC (10 μm) or E3 for 30 s. Then the irritant/E3 was washed out of the dish (1-min wash with ∼2–3 total volumes of fresh E3). Afterwards, the fish were transferred into a large Petri dish (∼138 mm in diameter) containing 100 ml of E3, and the positions of the 20 fish were subsequently recorded at various time points; this was done by taking a single photograph of all of the fish at each time point. The images were then analyzed using Image J (RRID:SCR_003070; Schneider et al., 2012), and the distance from the edge was determined for each of the 20 fish. We calculated the average distance from the dish’s edge for the 20 fish for each time point, and this average served as the measurement of thigmotaxis. For statistical purposes, we considered a dish average to be *n *=* *1.

#### Measurements of heart rate in restrained fish

Larval zebrafish, 5 dpf, were placed individually into a cell culture dish containing liquid 3% low melting point agarose and positioned to facilitate observation of heart rate. Once the agarose gelled, a dorsal area of the fish’s head was freed from the agarose to enable direct exposure of the skin to the AITC. The fish was then placed under a dissecting microscope and allowed to acclimate for 30 min. A baseline heart rate was determined by visual inspection for a period of 30 s. Thirty seconds after this baseline observation, AITC (10 μm) or E3 was added to the bath for 1 min. Another measurement of heart rate (30-s measurement period) was made 30 s after the onset of exposure to AITC/E3. The second measurement of heart rate was followed by a 1-min washout period in which the experimental solution was exchanged for fresh E3 using ∼2–3 total volumes of E3. Later, a final 30-s measurement of heart rate was made, or, in some cases, several 30-s postwashout measurements of heart rate were made. A similar protocol was followed for the experiments involving ruthenium red (RR) except that the RR-containing solution or E3 was washed into the bath 4 min before AITC application, or was washed into the bath as the AITC/E3 was being washed out.

### Experiments involving ibuprofen (IBU)

In the experiments using the anti-inflammatory drug IBU, the drug was present throughout every experiment. The IBU was dissolved in dimethylsulfoxide (DMSO) before dilution in E3 to a final concentration of 50 μm in 0.1% DMSO; the control solution was therefore E3 with 0.1% DMSO. All solutions used for AITC treatment or washout contained 50 μm IBU (0.1% DMSO) or E3 (0.1% DMSO), depending on the experimental condition. For example, when fish were treated with AITC, the experimental solution, depending on the condition, contained, in addition to 10 μm AITC, either 50 μm IBU (0.1% DMSO) or E3 (0.1% DMSO). Similarly, the solution used for washing out the AITC/E3 contained 50 μm IBU (0.1% DMSO) or E3 (0.1% DMSO). In all other respects the protocols used to assess the effect of IBU on AITC-induced changes in locomotion, thigmotaxis, and heart rate were identical to those described above.

### Imaging

To image AITC-induced changes in neuronal activity in the larval zebrafish brain, we used larvae (5–6 dpf) expressing GCAMP6s (Chen et al., 2013) under control of the *ELAV3* promoter (Vladimirov et al., 2014). A custom built, high-speed line scanning confocal microscope was initially used to observe large portions of the zebrafish brain (∼2.8 × 106 μm^3^) to identify brain regions areas whose activity correlated with behavioral changes induced by AITC. Initially, we focused on the hindbrain because previous studies showed this area was strongly activated by AITC (Randlett et al., 2015). Images of a volume (200 × 140 × 100 μm^3^; vol) of the hindbrain were recorded (5 vol/s 200 Hz) 1 min before, 1 min during, or 5 min after AITC/E3 application. Visual inspection of these recordings revealed an area that was strongly activated during AITC application and, importantly, whose neural activity persisted after the AITC was washed out (refer to Fig. 5*B*). This much smaller region (1075 μm^2^) was further investigated via standard confocal microscopy (488-nm excitation) using an LSM Pascal microscope (Zeiss) equipped with an inverter (LSM TECH). This microscope, although unable to record more than a limited region of the brain at one time, was configured for our experimental needs and was adequate to record neural activity from the area identified by the more powerful microscope. We restricted the region of interest to our identified area (1075 μm^2^) to enhance the recording speed (1.55 Hz) of the confocal microscope. After 30 min for acclimation, images (1-min recording) were taken to measure baseline neural activity. Five minutes after the baseline recording, 10 μm AITC or E3 was applied for 30 s and then washed out of the bath with fresh E3 for 1 min, after which images (1-min recording period) were again taken starting 3.5 min after the onset of AITC/E3 application. There was an increase in neural activity at the onset of the neural recording, which most likely reflected the animals’ response to the microscope’s laser; therefore, we only analyzed the last 30 s of the 1-min recording for both pretest and posttest images. We measured the mean fluorescence over this 30-s period and normalized this value to the pretest response (ΔF posttest/F pretest).

### Pharmacology

Sensitization was elicited with the chemical irritant AITC for 30 s to 1 min. To block TRP channels, we used RR (10 μm). IBU (50 μm) was used to mitigate inflammatory processes. AITC, IBU, and RR were purchased from Sigma; RR was also purchased from Tocris Bioscience.

### Statistical analyses

Statistical comparisons were conducted using unpaired *t* tests or ANOVAs. For experiments measuring behavior or heart rate in the same fish over time, repeated measures, between groups ANOVAs were used. Tukey’s HSD tests were used for all *post hoc* analyses.

## Results

### AITC, a chemical irritant, elicits a strong behavioral response in larval zebrafish

**Figure 1.**
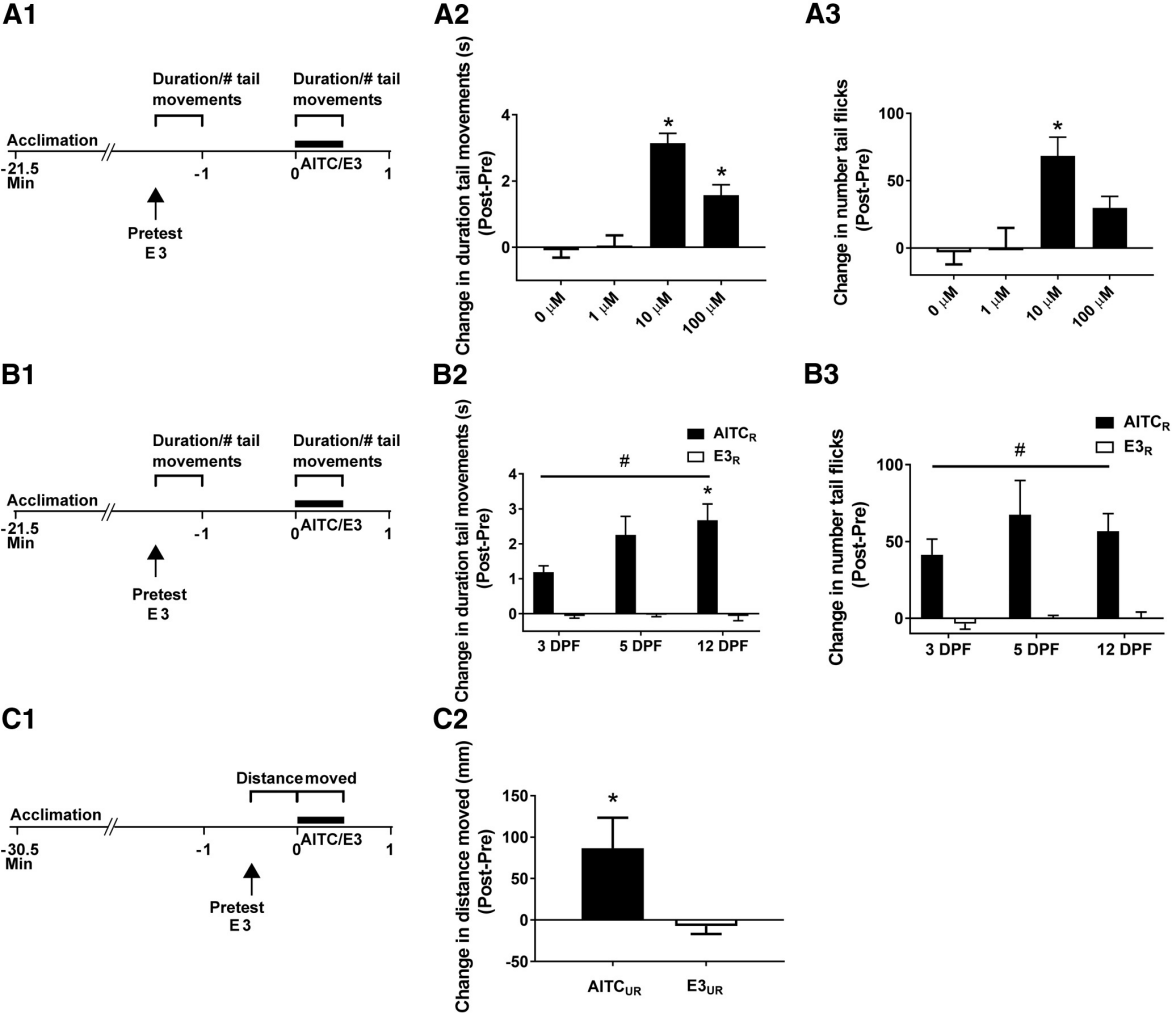
Locomotor activity in zebrafish larvae is enhanced in the presence of AITC. ***A1***, Protocol for the experiments presented in ***A2***, ***A3***. The duration of time of swimming-like tail movements and the number of tail flicks were measured in semi-restrained zebrafish (5–6 dpf) in the presence of AITC (30-s duration) or control solution (E3). During the pretest, given 1.5 min before application of AITC/E3, the response of the larva to application of E3 alone was measured. ***A2***, Change in the duration of tail movements in response to AITC/E3. A one-way ANOVA indicated that AITC significantly enhanced locomotion as measured by the duration of movements (*F*_(3,40)_ = 27.11; *p *<* *0.001). Tukey’s HSD *post hoc* tests indicated that fish treated with 10 μm (*n *=* *11) or 100 μm (*n *=* *11) AITC showed significantly more activity compared with fish that received either 0 μm (*n *=* *11) or 1 μm (*n *=* *11) AITC (*p *<* *0.05 for each comparison). ***A3***, Change in the number of tail flicks in response to AITC/E3. A one-way ANOVA indicated that AITC significantly increased the number of tail flicks produced by larvae (*F*_(3,40)_ = 7.85; *p *=* *0.0003). Tukey’s HSD *post hoc* tests indicated that fish treated with 10 μm AITC exhibited significantly more tail flicks than fish treated with either 0 or 1 μm AITC. Note that the results presented in ***A2***, ***A3*** are based on the same data. ***B1***, Experimental protocol for assessing the effect of development on AITC-induced alterations in locomotion. ***B2***, Change in duration of tail movements in response to AITC (10 μm, 30-s duration) in larvae of different ages. A two-way ANOVA examining the effect of developmental age and exposure to AITC revealed a significant interaction (*F*_(2,44)_ = 3.54; *p *=* *0.04) for change in duration of tail movements. For zebrafish at all developmental ages (AITC_RESTRAINED (_*_R_*_)_: 3 dpf, *n* = 8; 5 dpf, *n* = 7; 12 dpf, *n *=* *9; E3_R_: 3 dpf, *n* = 9; 5 dpf, *n* = 9; 12 dpf *n *=* *8) there was a main effect of enhanced locomotor response in response to 10 μm AITC (*F*_(1,44)_ = 77.82; *p *<* *0.001). In addition, Tukey’s HSD *post hoc* tests indicated that the 12-dpf group exhibited tail movements for significantly longer after exposure to AITC than did the 3-dpf group (*p *<* *0.05). ***B3***, Effect of AITC (10 μm) on tail flicks in zebrafish larvae of different ages. There was a significant main effect of exposure to the chemical irritant (*F*_(1,44)_ = 46.09; *p *<* *0.001). The interaction between AITC treatment and larval age was not significant (*F*_(2,44)_ = 0.60; *p *=* *0.55). (The results presented in ***B2***, ***B3*** are based on the same data.) ***C1***, Protocol for measuring the effect of AITC on locomotion in freely moving zebrafish larvae (5 dpf). ***C2***, AITC (10 μm, 30-s duration) produced an increase in distance moved (AITC_UNRESTRAINED (UR)_ group, *n *=* *12) compared with larvae exposed to control solution (E3_UR_ group, *n *=* *12), as indicated by an unpaired *t* test (*t*_(22)_ = 2.20; *p *=* *0.04). This figure shows means ± SEM; in addition, * indicates a significant (*p *<* *0.05) difference between groups and # indicates a significant (*p *<* *0.05) main effect.

AITC has previously been shown to elicit strong enhancement of locomotion in zebrafish larvae (Prober et al., 2008) and to substantially increase neural activity, particularly in the hindbrain (Randlett et al., 2015). We therefore investigated whether this substance might induce behavioral sensitization in larval zebrafish. To determine what concentrations of AITC might be effective in altering the behavior of larval zebrafish, we measured locomotor activity in semi-restrained larvae (Materials and Methods) in response to manually adding either E3 (control medium, 100 μl) or various concentrations of the irritant (100 μl of solution). We exposed semi-restrained larvae (5–6 dpf) to AITC or E3 and measured the subsequent change in duration of time spent making swimming-like tail movements (flicks) and in the number of tail flicks (Fig. 1*A*). Fish were initially stimulated with a head-directed ejection of E3 (100 μl) from a hand-held pipette (pretest); 1.5 min later the fish received a 30-s treatment with either AITC (1–100 μm final concentration in the bath) or fresh E3 (posttest). As previously shown by others (Prober et al., 2008; Randlett et al., 2015), we observed that concentrations of AITC ≥10 μm increased movement in the larvae as indicated by significant changes in both duration of swimming-like tail movements and number of tail flicks (Table 1).

**Table 1:** Normalized duration and number of tail flicks in 0–100 μm AITC in zebrafish larvae

AITC	Sample size (*n*)	Duration swimming; mean and SEM	Number of tail flicks; mean and SEM
0 μm	11	−0.08 ± 0.22 s	−3.18 ± 8.99
1 μm	11	0.05 ± 0.31 s	0.27 ± 14.69
10 μm	11	3.14 ± 0.30 s	68.45 ± 13.92
100 μm	11	1.57 ± 0.32 s	29.82 ± 8.51

To determine the age of onset of responsiveness to AITC in zebrafish, we exposed semi-restrained larvae between 3 and 12 dpf to AITC or E3 and measured the subsequent changes in tail movements (Fig. 1*B*). As before, larvae were first stimulated with E3 (100 μl) using a hand-held pipette (pretest) followed 1.5 min later by a 30-s treatment with either AITC (10 μm) or fresh E3 (posttest). A two-way ANOVA probing the developmental age of the fish and exposure to AITC revealed a significant interaction for change in duration of swimming-like movements (*p *=* *0.04), but not for the change in number of tail flicks (*p *=* *0.55). A Tukey’s HSD *post hoc* analysis revealed that older fish (12 dpf) exhibited a more prolonged period of tail movements in response to AITC than did younger fish (3 dpf; *p *<* *0.05). The main effect for exposure to AITC was significant (*p *<* *0.001) for both change in duration of tail movements and change in number of tail flicks (Table 2). Thus, fish of all developmental ages tested were responsive to AITC.

**Table 2:** Normalized duration and number of tail flicks in AITC or E3 from 3–12 dpf in zebrafish larvae

AITC_R_	Sample size (*n*)	Duration swimming; mean and SEM	Number of tail flicks; mean and SEM	E3_R_	Sample size (*n*)	Duration swimming; mean and SEM	Number of tail flicks; mean and SEM
3 dpf	8	1.19 ± 0.18 s	41.38 ± 10.13	3 dpf	9	−0.06 ± 0.06 s	−3.56 ± 3.56
5 dpf	7	2.26 ± 0.53 s	67.43 ± 22.36	5 dpf	9	−0.03 ± 0.06 s	0.00 ± 1.83
12 dpf	9	2.67 ± 0.47 s	56.78 ± 11.39	12 dpf	8	−0.07 ± 0.13 s	−0.63 ± 3.40

To confirm that locomotion in freely moving fish is similarly affected by AITC, we measured the distance moved by 5-dpf fish unrestrained in agarose during an initial 30-s period with the fish in E3 (pretest) and during a subsequent 30-s exposure to 10 μm AITC or fresh E3 (posttest; Fig. 1*C*). Freely moving larvae moved a greater distance in the presence of the irritant (AITC_UR_ group = 79.88 ± 37.84 mm) than in the control solution (E3_UR_ group = −6.09 ± 9.75 mm; *p *<* *0.05). AITC therefore increased movement in both semi-restrained and freely moving larvae.

### Exposure to AITC appears to sensitize locomotion

**Figure 2.**
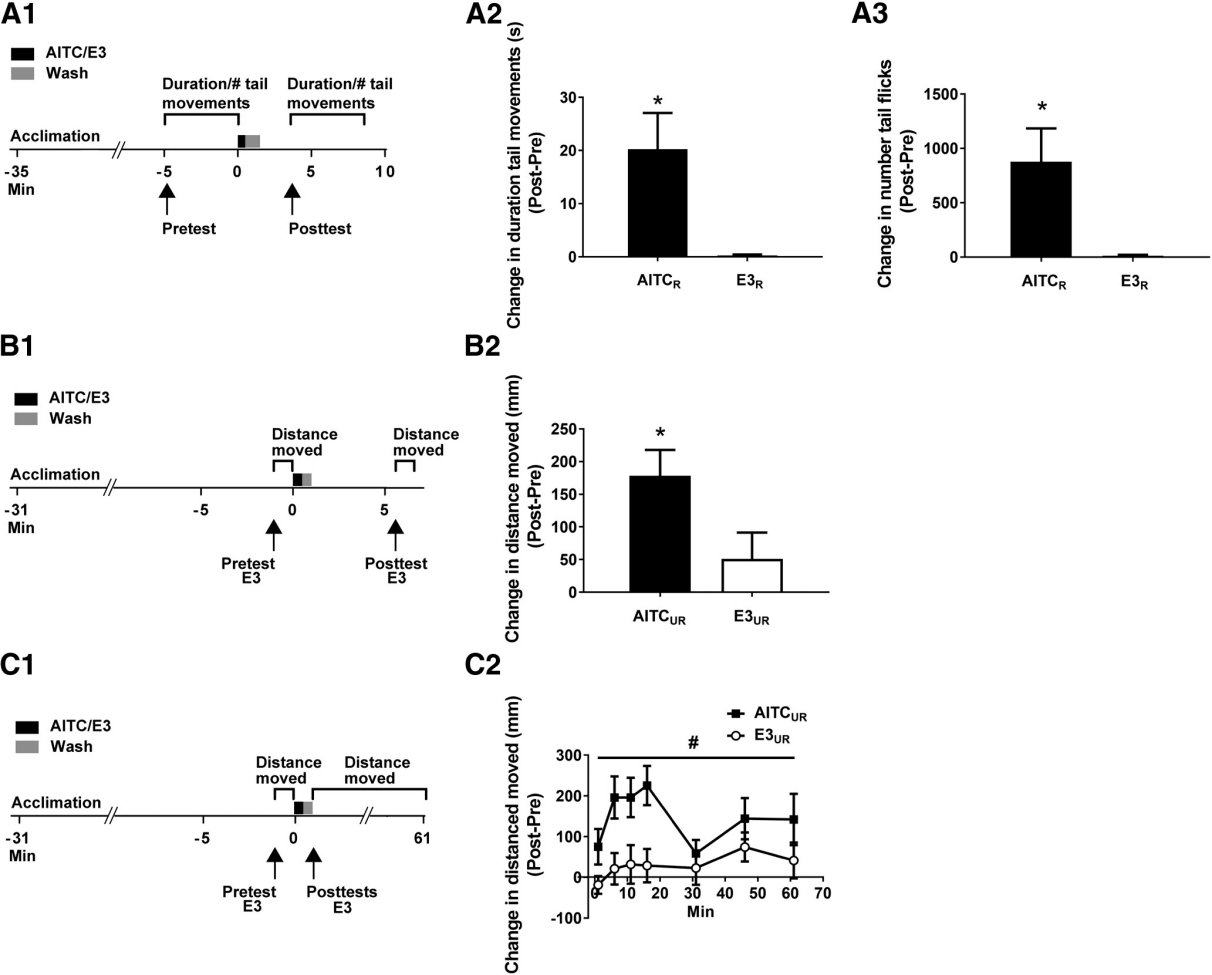
AITC elicits persistently enhanced locomotion in zebrafish larvae. ***A1***, Experimental protocol for tests of sensitization-like enhancement of locomotor activity in semi-restrained larvae. The movement of the larvae, either swimming-like behavior or tail flicks, was sampled during the 5-min period immediately before the onset of AITC/E3 exposure (30 s), as well as during the period 2–7 min after a 1-min washout of the drug/E3. ***A2***, AITC caused an increase in swimming-like tail movements that persisted for ≥5 min. An unpaired *t* test indicated that fish treated with AITC (AITC_R_ group, *n *=* *12) moved for a longer time after the AITC was washed out than did fish treated with E3 alone (E3_R_ group, *n *=* *12; *t*_(22)_ = 2.94, *p *=* *0.008). ***A3***, AITC also caused a persistent increase in the number of spontaneous tail flicks. AITC-exposed fish exhibited significantly more tail flicks following washout of the irritant than fish exposed to E3 alone (*t*_(22)_ = 2.83, *p *=* *0.01). Note that the results presented in ***A2***, ***A3*** are based on the same data. ***B1***, Experimental protocol for testing whether AITC had a persistent effect on locomotion in freely swimming larvae. ***B2***, Distance moved by unrestrained larvae in response to AITC/E3. The total distance moved was measured for the 60 s immediately preceding the onset of a 30-s treatment with AITC/E3 and during the period 4.5–5.5 min after washout (30 s long) of the drug/E3. The change in distance moved by larvae in response to AITC (AITC_UR_ group, *n *=* *10) was significantly greater than that by larvae exposed simply to E3 (E3_UR_ group, *n *=* *10; *t*_(18)_ = 2.33; *p *=* *0.03). ***C1***, Experimental protocol for determining the persistence of AITC’s enhancement of locomotion in freely swimming larvae. ***C2***, Change in distance moved by larvae in response to AITC/E3 over a 60-min time period. For this purpose, the total distance moved was measured for the 60 s immediately preceding the onset of AITC/E3 treatment (30-s duration) and periodically over 60 min after washout (30 s) of AITC/E3. A repeated-measures, two-way ANOVA failed to find a significant interaction (*F*_(6,108)_ = 1.50; *p *=* *0.19). However, the main effect for exposure to AITC or E3 was significant (*F*_(1,18)_ = 7.47; *p *=* *0.01), indicating that the change in distance moved by larvae following delivery of AITC (AITC_UR_ group, *n *=* *10) was significantly greater than that by larvae after exposure to E3 alone (E3_UR_ group, *n *=* *10). A repeated-measures, two-way ANOVA using non-normalized data found a significant interaction (*F*_(7,126)_ = 2.12; *p *<* *0.05). Probes of this interaction using one-way ANOVAs indicated significant differences at the 6-min (*F*_(1,18)_ = 8.60, *p *=* *0.009), 11-min (*F*_(1,18)_ = 9.10, *p *=* *0.007), and 16-min (*F*_(1,18)_ = 15.09, *p *=* *0.001) tests between the AITC_THIGMO_ (*n* = 10) and E3_THIGMO_ (*n *=* *10) groups. This figure shows means ± SEM, with * indicating a significant (*p *<* *0.05) difference between groups and # indicating a significant (*p *<* *0.05) main effect.

Exposure to aversive stimuli such as electrical shocks, strong tactile stimulation, and odorants causes sensitization of behavioral responses, a nonassociative form of learning and memory, in a range of organisms (Thompson and Spencer, 1966; Carew et al., 1971; Hebb et al., 2003). To determine whether the alterations of the behavioral responses induced by AITC (Fig. 1) persisted after removal of this aversive agent, thereby indicating sensitization of the responses, we measured locomotor activity after AITC was washed out from the bathing solution. Using semi-restrained larval zebrafish, we measured spontaneous swimming-like tail movements during a 5-min recording period (pretest) during which a larva was bathed in E3. Immediately after the pretest the larva was given a 30-s exposure to either 10 μm AITC or E3, after which the AITC/E3 was washed out with fresh E3 for 1 min; after a 2-min wait period movements of the fish were recorded for the next 5 min (posttest; Fig. 2*A1*). Both the duration of tail movements (AITC_R_ group = 20.23 ± 6.80 s) and the number of tail flicks (AITC_R_ group = 876.50 ± 304.82) were significantly greater in zebrafish larvae following exposure to AITC than after exposure to E3 (E3_R_ group, duration of swimming = 0.25 ± 0.18 s; and number of tail flicks = 12.33 ± 8.88; Fig. 2*A2*).

Next, we tested whether AITC exposure sensitizes locomotion in unrestrained, freely moving fish. Accordingly, we measured the distance the fish moved during a 1-min period with the fish in E3 (pretest; Fig. 2*B1*). Immediately afterward the fish were exposed for 30 s to AITC (10 μm) or E3. The AITC/E3 was replaced with fresh E3 (30-s wash) and then 5.5 min after the onset of exposure to AITC/E3 the distance moved by the fish during a 1-min observation period was measured (posttest). The distance moved was significantly enhanced in the AITC-treated fish (AITC_UR_ group, difference in distance moved from pretest to posttest = 174.67 ± 35.36 mm) compared with the E3-treated group (E3_UR_ group, difference in distance moved from pretest to posttest = 50.98 ± 39.46 mm; *p *<* *0.05; Fig. 2*B2*). Thus, AITC appears to sensitize freely moving, as well as restrained, larvae.

**Figure 3.**
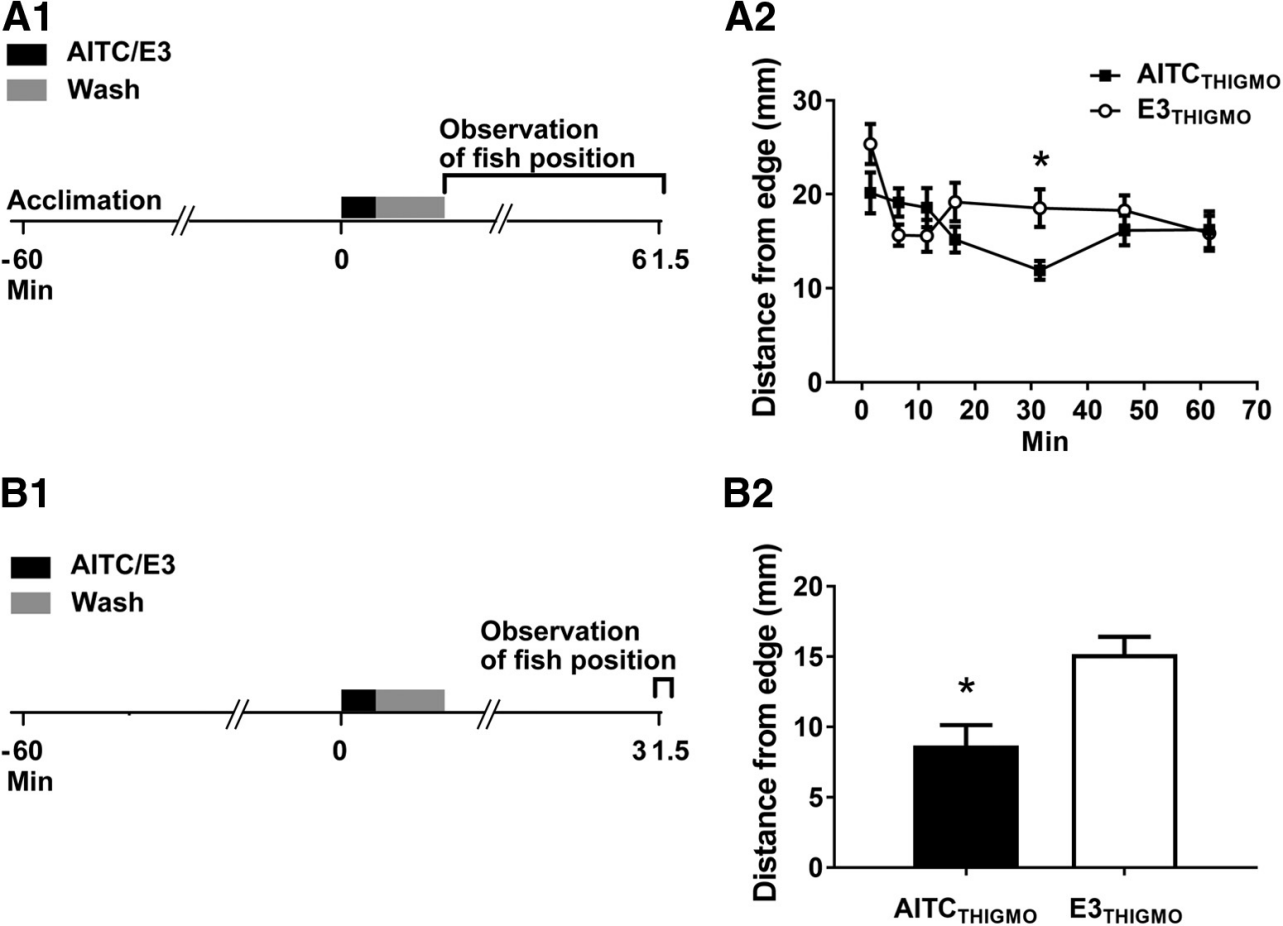
Thigmotaxis is enhanced by AITC in larval zebrafish. ***A1***, Experimental protocol. The larvae, 20 at a time, were placed into a Petri dish and allowed to acclimate to the dish for 60 min, after which they were exposed to AITC/E3 for 30 s. Following 1 min of washout of the AITC/E3, the larvae were transferred to a larger Petri dish and their positions measured over time. ***A2***, Postexposure effect of AITC on thigmotaxis. A repeated-measures, two-way ANOVA (*F*_(6,108)_ = 3.30; *p *=* *0.01) found an interaction between exposure to AITC/E3 and the time of test. Probes of this interaction using one-way ANOVAs indicated that only for 31.5-min test was there a significant difference between the AITC_THIGMO_ (*n* = 10) and E3_THIGMO_ (*n *=* *10) fish (*F*_(1,18)_ = 8.91, *p *=* *0.008). ***B1***, Experimental protocol for planned 31.5-min test of AITC-induced increase in thigmotaxis. ***B2***, Effect of AITC/E3 on thigmotaxis at 31.5 min posttreatment. Following exposure to AITC, fish (AITC_THIGMO_ group*, n *=* *8) were significantly closer to the edge of the dish than were fish after exposure to E3 alone (E3_THIGMO_ group, *n *=* *8; *t*_(14)_ = 3.40, *p *=* *0.004). This figure shows means ± SEM; * indicates a significant (*p *<* *0.05) difference between groups.

**Figure 4.**
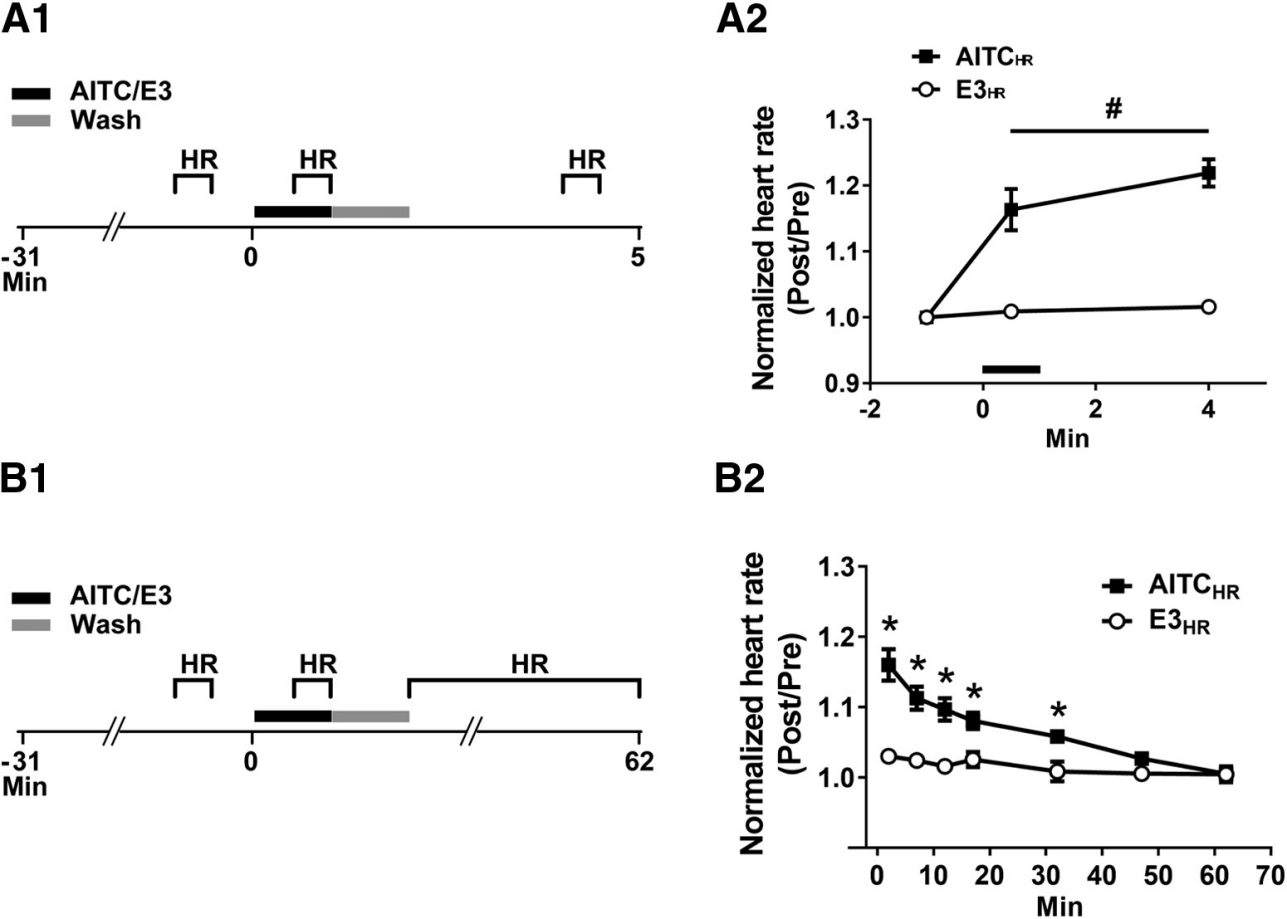
Heart rate in larval zebrafish is persistently increased by AITC. ***A1***, Experimental protocol for examining the effect of AITC/E3 on heart rate. The experiments were performed on larvae fully restrained in agar. Heart rate was measured by visual inspection during 30-s periods that began at: 1 min before the onset of treatment (for 1 min) with AITC/E3; 30 s after the onset of AITC/E3 treatment; and 2 min after the end of the 1-min washout period. ***A2***, Effect of AITC/E3 on larval heart rate. A repeated-measures, two-way ANOVA revealed a significant overall effect of AITC exposure (*F*_(1,14)_ = 78.68, *p *<* *0.001). The AITC-exposed fish (AITC_HR_ group, *n *=* *8) showed an increase in normalized heart rate in the presence of the chemical irritant as well as at 2 min after washout compared with fish (E3_HR_ group, *n *=* *8) exposed only to E3. ***B1***, Experimental protocol to determine the persistence of the increase in heart rate caused by AITC. ***B2***, Postexposure effect of AITC on heart rate. A repeated-measures, two-way ANOVA (*F*_(6,84)_ = 14.44; *p *=* *0.001) found an interaction between exposure to AITC/E3 and the time of test. Probes of this interaction using one-way ANOVAs indicated that the differences between the AITC-treated (*n *=* *8) and the E3-treated (*n *=* *8) groups were significant (*p *<* *0.05) on the tests from immediately after washout of AITC/E3 up to and including the 32-min test. This figure shows means ± SEM, with * indicating a significant (*p *<* *0.05) difference between groups and # indicating a significant (*p *<* *0.05) main effect.

To determine the length of the sensitization memory for locomotion, we used methods like those in the experiments presented in Figure 2*B*. After recording distance moved during a 1-min pretest period, we exposed the fish to AITC (10 μm) or E3 for 30 s and washed out these solutions for a 30-s period of time. We measured the distance moved for 1-min periods 1 min after the onset of exposure to AITC/E3 and at 6, 11, 16, 31, 46, and 61 min (Fig. 2*C*). A repeated-measures, two-way ANOVA was used to define the period of time that AITC enhanced locomotion. The interaction between exposure condition and time of testing was not significant (*p *=* *0.18); however, the main effect for the presence or absence of AITC was significant (*p *< 0.05). Therefore, locomotion in the AITC-treated group was enhanced compared with the E3-treated group for up to 60 min after washout of AITC/E3 (Table 3). The lack of a significant interaction did not permit a more fine-grain temporal resolution of the duration of the effect of sensitization on locomotion; however, we repeated the analysis using the non-normalized (raw) data. A repeated-measures two-way ANOVA performed on the raw data did reveal a significant interaction (*p *<* *0.05). We probed this interaction with one-way ANOVAs across all time points. We observed significant differences at the 6-, 11-, and 16-min time points (*p *<* *0.05); all other time points, including the pretest, failed to reach significance (Table 4). This analysis suggests that the sensitization of locomotion persisted for at least 16 min, but <31 min, consistent with the effect of AITC exposure on other behaviors/physiological processes (Tables 5, 6; Figs. 3, 4).

**Table 3:** Time course of the AITC-induced sensitization of locomotion in zebrafish larvae (normalized data)

AITC_UR_ group (*n *=* *10)	Mean and SEM	E3_UR_ group (*n *=* *10)	Mean and SEM
1 min	75.00 ± 43.90 mm	1 min	−18.54 ± 21.81 mm
6 min	196.30. ± 51.79 mm	6 min	21.25 ± 38.85 mm
11 min	196.24 ± 48.53 mm	11 min	31.97 ± 47.35 mm
16 min	225.57 ± 47.94 mm	16 min	28.87 ± 40.93 mm
31 min	58.87 ± 32.95 mm	31 min	22.91 ± 41.01 mm
46 min	144.25 ± 50.83 mm	46 min	74.56 ± 35.78 mm
61 min	142.31 ± 63.21 mm	61 min	41.61 ± 44.19 mm

**Table 4:** Time course of the AITC-induced sensitization of locomotion in zebrafish larvae (raw/non-normalized data)

AITC_UR_ group (*n *=* *10)	Mean and SEM	E3_UR_ group (*n *=* *10)	Mean and SEM
Pretest	114.03 ± 39.27 mm	Pretest	105.95 ± 30.17 mm
1 min	189.03 ± 50.28 mm	1 min	87.41 ± 32.92 mm
6 min	310.33 ± 52.55 mm	6 min	127.21 ± 33.72 mm
11 min	310.27 ± 40.40 mm	11 min	137.92 ± 40.41 mm
16 min	339.60 ± 32.91 mm	16 min	134.82 ± 41.18 mm
31 min	172.90 ± 42.95 mm	31 min	128.86 ± 40.53 mm
46 min	258.28 ± 49.34 mm	46 min	180.51 ± 42.60 mm
61 min	256.34 ± 60.48 mm	61 min	147.56 ± 42.24 mm

**Table 5:** Time course of the AITC-induced sensitization of thigmotaxis in zebrafish larvae

AITC_THIGMO_ group (*n *=* *10)	Mean and SEM	E3_THIGMO_ group (*n *=* *10)	Mean and SEM
1.5 min	18.55 ± 1.90 mm	1.5 min	23.33 ± 1.97 mm
6.5 min	17.60 ± 1.30 mm	6.5 min	14.41 ± 1.04 mm
11.5 min	17.11 ± 1.81 mm	11.5 min	14.35 ± 1.90 mm
16.5 min	13.97 ± 1.21 mm	16.5 min	17.66 ± 1.90 mm
31.5 min	10.96 ± 0.87 mm	31.5 min	17.05 ± 1.85 mm
46.5 min	14.87 ± 1.38 mm	46.5 min	16.83 ± 1.48 mm
61.5 min	14.95 ± 1.70 mm	61.5 min	14.59 ± 1.71 mm

**Table 6:** Time course of the AITC-induced sensitization of heart rate in zebrafish larvae

AITC_HR_ group (*n *=* *8)	Mean and SEM	E3_HR_ group (*n *=* *8)	Mean and SEM
2 min	1.160 ± 0.022 BPM	2 min	1.030 ± 0.010 BPM
7 min	1.113 ± 0.017 BPM	7 min	1.024 ± 0.006 BPM
12 min	1.097 ± 0.016 BPM	12 min	1.016 ± 0.009 BPM
17 min	1.081 ± 0.012 BPM	17 min	1.026 ± 0.011 BPM
32 min	1.058 ± 0.009 BPM	32 min	1.008 ± 0.014 BPM
47 min	1.027 ± 0.010 BPM	47 min	1.005 ± 0.010 BPM
62 min	1.005 ± 0.007 BPM	62 min	1.005 ± 0.011 BPM

### AITC exposure increases thigmotaxis in larval zebrafish

Thigmotaxis, the propensity of an organism to move away from the center of an open area, is considered a measure of anxiety in animals and humans (Christmas and Maxwell, 1970; Prut and Belzung, 2003; Schnörr et al., 2012; Ahmad and Richardson, 2013; Walz et al., 2016). To assess the effect of AITC on thigmotaxis in larval zebrafish, unrestrained larvae (5 dpf) in a small Petri dish were exposed to either 10 μm AITC or E3 for 30 s, after which the AITC/E3 was washed out with fresh E3 for 1 min. Then the fish were rapidly transferred to a larger Petri dish (20 fish per dish), and each fish’s distance from the edge of the dish was measured at 1.5, 6.5, 11.5, 16.5, 31.5, 46.5, and 61.5 min after the onset of the exposure to AITC/E3 (Fig. 3*A1*). From these data, a group mean position of the 20 larvae was calculated for each time (Fig. 3*A2*). A repeated-measures, two-way ANOVA revealed a significant interaction (*p *<* *0.05). A one-way ANOVA indicated that AITC increased thigmotactic behavior at the 30-min test (AITC, mean distance from edge = 10.96 ± 0.87 mm; E3, mean distance from edge = 17.05 ± 1.85 mm, *p *<* *0.05). No significant differences were observed between AITC-treated fish and E3-treated fish at any other time point (Table 5). Thus, AITC causes a short-lived (<45 min) increase in thigmotaxis the onset of which requires ∼30 min.

To confirm the finding of significant thigmotaxis at 30 min after exposure to AITC, we replicated this experiment, measuring thigmotaxis only at 30 min postwashout; otherwise, the protocol was identical to that in the experiments presented in Figure 3*A*. As shown in Figure 3*B*, there was a significant increase in thigmotaxis in the AITC-treated group (AITC_THIGMO_ group = 7.97 ± 1.34 mm) compared with the group treated only with E3 (E3_THIGMO_ group = 13.96 ± 1.15 mm; *p *<* *0.05) at the planned 30-min test.

### AITC-induced behavioral sensitization involves activation of the autonomic nervous system and depends on TRP channels

Induction of behavioral sensitization often involves activation of the autonomic nervous system (Krontiris-Litowitz, 1999; Bouwknecht et al., 2000). Accordingly, we investigated whether AITC exposure activates the sympathetic nervous system in larval zebrafish; we used heart rate as an indicator of autonomic nervous system activation. Heart rate was measured in larvae fully restrained in agarose before, during a 1-min treatment with either AITC (10 μm) or E3, and after the AITC/E3 was washed out of the holding dish. The normalized heart rate of larvae was significantly enhanced in the presence of AITC [AITC_HR_ group = 1.164 ± 0.031 beats per minute (BPM)], as well as at the 4-min test (∼2 min after the irritant was washed out of the bath; AITC_HR_ group = 1.219 ± 0.021 BPM) compared with a group of larvae exposed only to E3 (E3_HR_ group, initial measurement = 1.009 ± 0.003 BPM; measurement at the 4-min test = 1.016 ± 0.009 BPM; Fig. 4*A2*). As these data indicate, the 1-min exposure to AITC induced short-term sensitization of heart rate in zebrafish larvae. To determine how long heart rate remained elevated following AITC exposure, we used methods like those in the experiments presented in Figure 4*A*. Restrained fish were exposed to 10 μm AITC or E3 for 1 min. We measured heart rate 1 min before the onset of the AITC/E3 treatment and washout procedures (1 min), as well as at 2, 7, 12, 17, 32, 47, and 62 min after the onset of the AITC/E3 (30-s observation period throughout; Fig. 4*B*). A repeated-measures, two-way ANOVA revealed a significant interaction (*p *<* *0.05), and this interaction was therefore probed with one-way ANOVAs at each time point. Heart rate was significantly increased (*p *<* *0.05) in AITC-treated animals compared with E3-treated animals during the 2- through 32-min observation periods (Table 6). There were no significant (*p *>* *0.05) differences between the AITC- and E3-treated groups during the 47- and 62-min observation periods. Thus, heart rate remained sensitized in larvae after AITC exposure for at least 32 min but not longer than 47 min.

**Figure 5.**
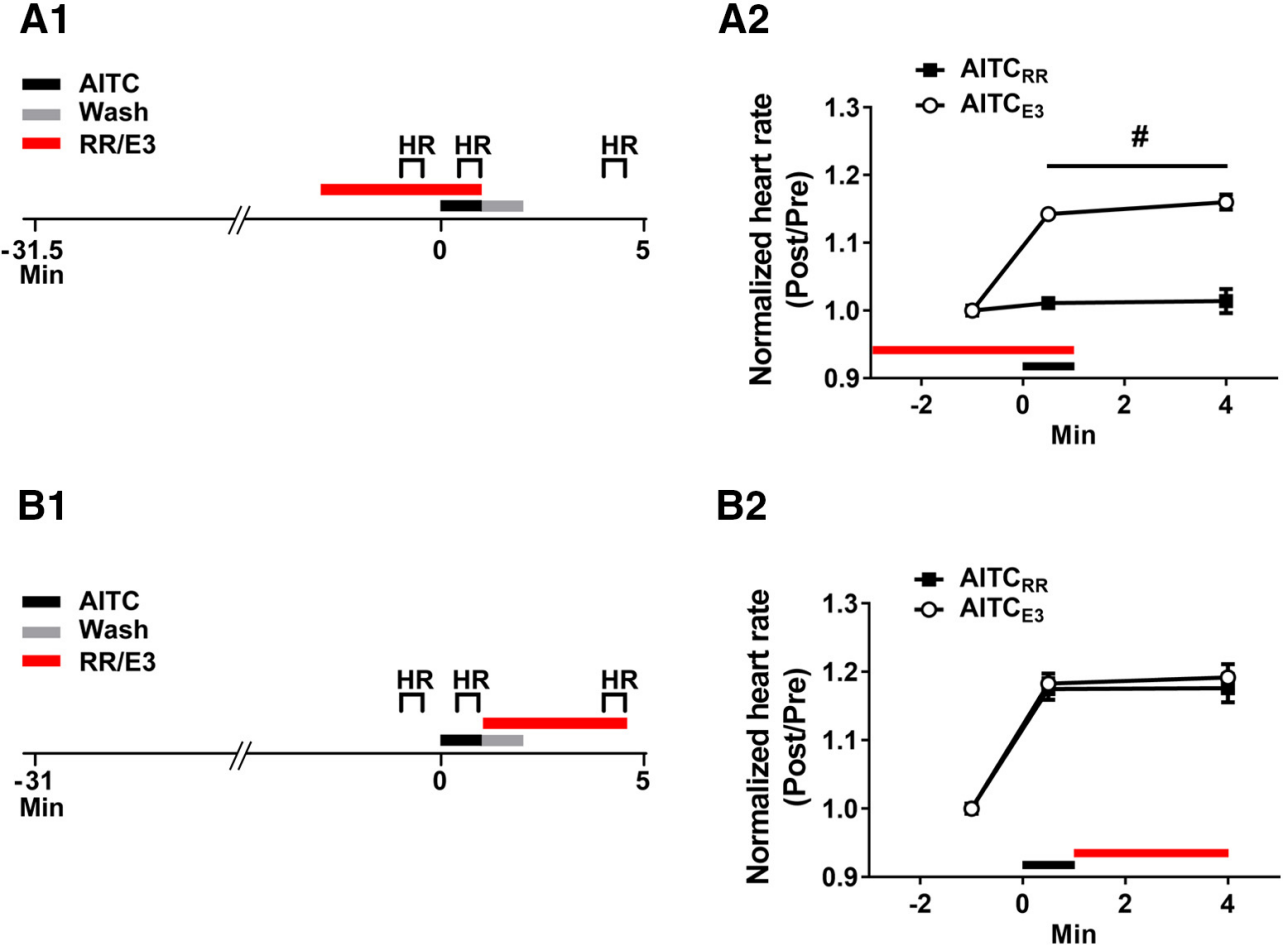
An antagonist of TRP channels blocks the increase in larval heart rate observed during the presence of AITC, but not that observed after washout of the irritant. ***A1***, Experimental protocol for the test of the effect of RR (10 μm) on AITC-elicited change in heart rate. RR was present in the bath for 4 min before the onset of a 1 min-long exposure to AITC/E3. After treatment with AITC/E3, the RR was washed out of the holding dish together with the chemical irritant/E3, and the bathing solution was replaced with fresh E3. The heart rate of the larva was measured for 30-s periods beginning: 1 min before the onset of AITC/E3; 30 s after the onset of AITC/E3; and 2 min after the end of washout (1 min in duration). ***A2***, Effect of RR on the prolonged AITC-induced increase in heart rate. A repeated-measures, two-way ANOVA indicated that fish exposed to RR before and during AITC application (AITC_RR_ group, *n *=* *8) exhibited a significantly lower heart rate than did fish (AITC_E3_ group, *n *=* *8) not exposed to RR, both when AITC was present in the bath and for ≤3 min after washout of the irritant (*F*_(1,14)_ = 67.06, *p *=* *0.001). ***B1***, Experimental protocol for the test of RR’s effect on the AITC-induced heart rate increase when the TRP receptor antagonist was applied following washout of AITC. The RR was applied to the bath for a 3.5-min period beginning at the start of washout of AITC/E3. The heart rate was measured during three 30-s periods that began: 1 min before the onset of AITC/E3; 30 s after the onset of AITC/E3; and 2 min after the end of washout. ***B2***, Effect of RR on the enhancement of larval heart rate elicited by AITC if RR was only present in the bath after washout of AITC. A repeated-measures, two-way ANVOA indicated that the heart rate of larvae when RR was applied after AITC treatment (AITC-RR, *n *=* *8) did not differ significantly from that of AITC-treated larvae not exposed to RR (AITC-E3, *n *=* *8; *F*_(1,14)_ = 0.35, *p *=* *0.56). This figure shows means ± SEM; # indicates a significant (*p *<* *0.05) main effect.

Previous work found that AITC activates TRP channels expressed on the trigeminal and Rohon Beard sensory neurons in larval zebrafish (Prober et al., 2008). To confirm that AITC-induced behavioral sensitization was because of activation of TRP channels, we used RR, which antagonizes these receptors in zebrafish (Prober et al., 2008; Fig. 5). Bath application of RR (10 μm) for 4 min before AITC exposure blocked the increase in the normalized heart rate in the presence of the irritant (AITC_RR_ group = 1.011 ± 0.010 BPM; AITC_E3_ group = 1.143 ± 0.009 BPM), as well as the persistent elevation of heart rate observed after washout of AITC (4-min test: RR-AITC group = 1.014 ± 0.018 BPM; E3-AITC group = 1.160 ± 0.011 BPM; Fig. 5*A2*).

Possibly, the apparent sensitization of heart rate because of treatment with AITC was because of incomplete washout; alternatively, TRP channel activation might have caused a persistent sensory response in the absence of AITC (but see Hinman et al., 2006). To rule out these potential explanations for the apparent sensitization shown in Figure 5*A*,*B*, we performed another experiment in which RR was added to the holding dish after exposure to AITC (Fig. 5*B1*). Because RR antagonizes AITC-induced activation of TRP channels even when the irritant is applied before the onset of RR treatment (Prober et al., 2008), this protocol should prevent any prolonged sensory response to AITC after its ostensible washout. Application of RR following the ostensible washout of AITC did not block the increase in heart rate produced by the irritant (AITC-E3 group: heart rate during AITC treatment = 1.183 ± 0.015 BPM; 4-min test = 1.192 ± 0.019 BPM vs AITC-RR group: heart rate during AITC treatment = 1.175 ± 0.016 BPM; 4-min test = 1.176 ± 0.021 BPM). Thus, the persistent elevation of heart rate following exposure to AITC cannot be attributed to either incomplete washout of this aversive agent or to sustained activity of sensory neurons resulting from prolonged TRP channel activation.

### The anti-inflammatory drug IBU failed to reduce the behavioral sensitization caused by AITC exposure

AITC and other TRPA1 agonists have been shown to induce pain and activate inflammatory processes (Bautista et al., 2006; Prober et al., 2008; Moilanen et al., 2012; Curtright et al., 2015; Esancy et al., 2018); these findings suggest an alternative explanation for the behavioral enhancements we observed. Some inflammatory responses and behavioral changes induced by TRP1 agonists are sensitive to anti-inflammatory agents (Moilanen et al., 2012; Ellis et al., 2018); others, however, are unaffected by IBU, indicating that they are independent of IBU-sensitive anti-inflammatory processes (Curtright et al., 2015). We asked whether the persistent AITC-induced behavioral changes we observed result from inflammation or, instead, represent nonassociative memory induced by learning-related neuroplastic changes (Walters et al., 1991; Walters and Ambron, 1995). IBU has been shown to effectively block inflammatory processes (Bancos et al., 2009; Moilanen et al., 2012) and is effective in zebrafish larvae (Ellis et al., 2018); we therefore tested whether IBU could ameliorate or block any of the AITC-induced behavioral or physiological changes we observed (Figs. 2-4) by exposing zebrafish to either 50 μm IBU, twice the concentration used by Ellis et al. (2018) to minimize IBU-sensitive inflammation, or the vehicle solution alone (0.1% DMSO in E3) for 30 or 60 min before, and during, experimental manipulations. The drug or vehicle control was maintained in the bath throughout the experiment.

**Figure 6.**
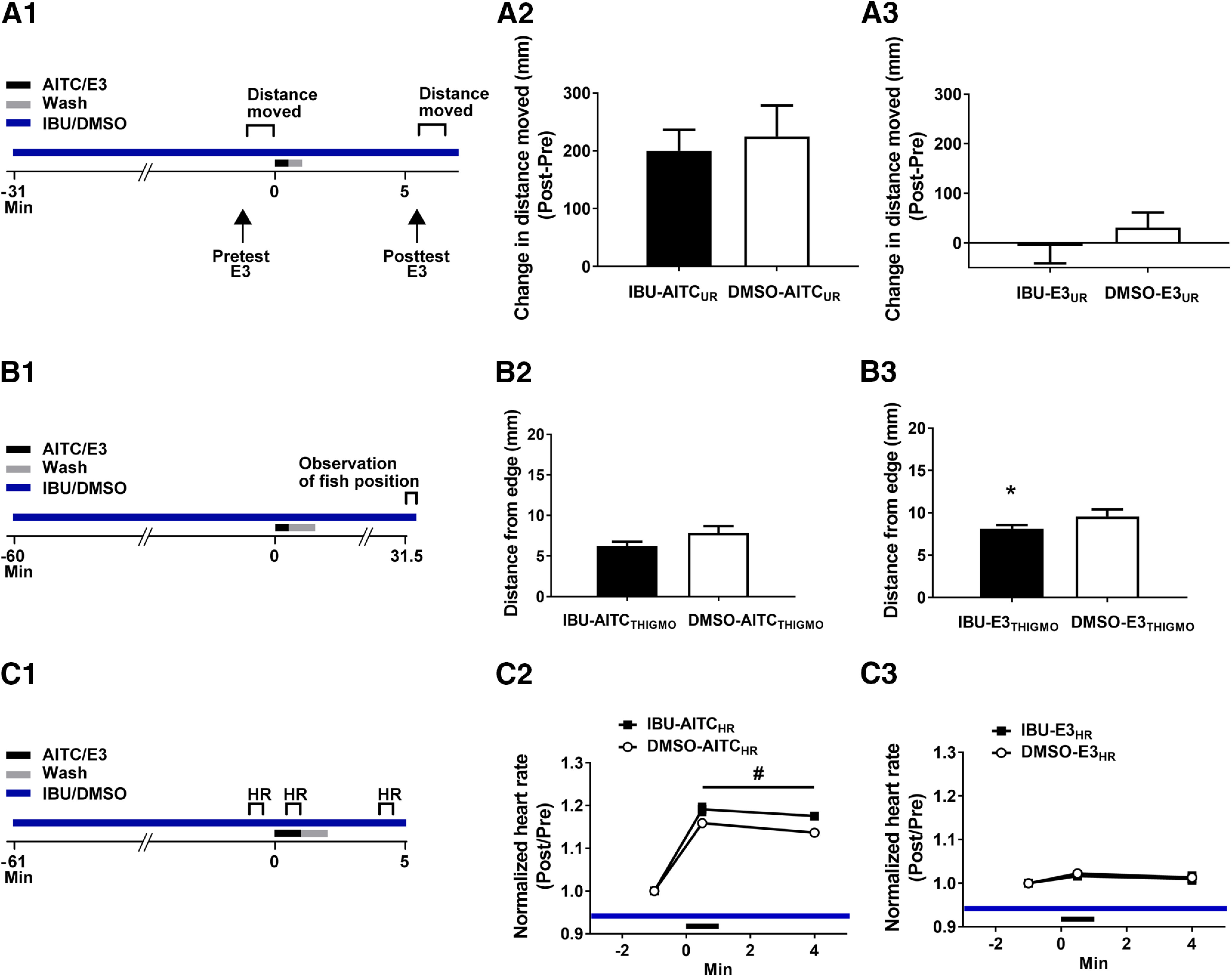
IBU does not block the enhancement of locomotion, thigmotaxis, and heart rate caused by AITC in larval zebrafish. ***A1***, Experimental protocol to test the effects of IBU on locomotion in freely moving larvae. ***A2***, The groups exposed to AITC (10 μm, 30-s duration; IBU-AITC_UR_ group, *n *=* *10; DMSO-AITC_UR_ group, *n *=* *10) did not differ significantly in the distance moved, regardless of whether or not IBU was present in the bath, as indicated by an unpaired *t* test (*t*_(18)_ = 0.39; *p *=* *0.70). ***A3***, The presence of IBU did not change the distance of movement in the groups (IBU-E3_UR_ group, *n *=* *10; DMSO-E3_UR_ group, *n *=* *10) that were treated with E3 for 30 s rather than the irritant. The difference between the two groups was nonsignificant (unpaired *t* test: *t*_(18)_ = 0.79, *p *=* *0.44). ***B1***, Experimental protocol used to test the effects of IBU on thigmotaxis. ***B2***, Effect of IBU/DMSO on thigmotaxis at 31.5 min after the onset of treatment with AITC/E3 (30-s duration). Thigmotaxis in fish placed into IBU and AITC (IBU-AITC_THIGMO_ group, *n *=* *18) was indistinguishable from that of fish placed in DMSO and AITC (DMSO-AITC_THIGMO_ group, *n *=* *20; *t*_(36)_ = 1.04, *p *=* *0.31). ***B3***, Effect of IBU/DMSO on thigmotaxis at 30 min after treatment with E3. Fish placed in IBU and then exposed for 30 s to (fresh) E3 (IBU-E3_THIGMO_, *n *=* *20) exhibited greater thigmotaxis than did fish treated identically except for being placed in DMSO before E3 exposure (DMSO-E3_THIGMO_ group, *n *=* *20*; t*_(38)_ = 2.42, *p *=* *0.02). ***C1***, Experimental protocol used to examine the effect of IBU on the increase in larval heart rate caused by AITC. ***C2***, Effect of IBU/DMSO on the AITC-induced alteration in heart rate. A repeated-measures, two-way ANOVA revealed a significant overall effect of drug exposure (*F*_(1,10)_ = 6.89, *p *=* *0.03). The fish placed in IBU before AITC exposure (IBU-AITC_HR_ group, *n *=* *6) exhibited a faster normalized heart rate while the chemical irritant was present in the bath, as well as at 2 min after washout of AITC, than did fish placed in DMSO before being treated with AITC (DMSO-AITC_HR_ group, *n *=* *6). ***C3***, Effect of IBU/DMSO on baseline larval heart rate. A repeated-measures, two-way ANOVA revealed no significant effect of drug exposure (*F*_(1,8)_ = 0.19, *p *=* *0.67). Neither the fish initially immersed in IBU-containing solution (IBU-E3_HR_ group, *n *=* *5) nor the fish initially immersed in the vehicle (DMSO-E3_HR_ group, *n *=* *5) showed any alterations in normalized heart in response to treatment with E3. This figure shows means ± SEM, with * indicating a significant (*p *<* *0.05) difference between groups and # indicating a significant (*p *<* *0.05) main effect.

First, we examined whether IBU reduced the prolonged locomotion we observed after treatment with AITC. We measured the distance the unrestrained fish moved during a 1-min period (pretest) that began 1 min before the onset of a 30-s exposure to AITC (10 μm)/E3 in the presence or absence of IBU (Fig. 6*A1*). (The AITC/E3 was washed out for 30 s following the treatment with the irritant/control vehicle. During washout, IBU or DMSO alone was washed back into the bath.) The distance moved by the fish during a 1-min posttest beginning 5.5 min after the onset of the AITC treatment was also measured. As shown in Figure 6*A2*, the distance moved after AITC exposure was not significantly different between the group exposed to IBU (IBU-AITC_UR_ group, difference in distance moved from pretest to posttest = 200.15 ± 36.11 mm) and the DMSO-treated group (DMSO-AITC_UR_ group, difference in distance moved from pretest to posttest = 225.01 ± 53.46 mm; *p *=* *0.70). Furthermore, IBU did not appear to induce nonspecific changes in locomotion: the group not exposed to AITC but treated with IBU (IBU-E3_UR_ group, difference in distance moved from pretest to posttest = −5.92 ± 35.13 mm) was not significantly different from the AITC-untreated group exposed to 0.1% DMSO (DMSO-E3_UR_ group, difference in distance moved from pretest to posttest = 30.93 ± 30.26 mm, *p *=* *0.44; Fig. 6*A3*). Taken together, these data indicate that the observed behavioral enhancement in locomotion was unlikely to have resulted from inflammatory processes.

Similarly, we tested whether the persistent thigmotaxis observed after exposure to AITC could be reduced by IBU. Larvae were exposed to either 50 μm IBU or 0.1% DMSO for 1 h; they were then treated with 10 μm AITC/E3 in 50 μm IBU/0.1% DMSO for 30 s, after which the AITC was washed out and IBU or DMSO was washed in for 1 min. Then the larvae were rapidly transferred to a larger Petri dish (20 fish per dish) containing IBU or DMSO, and each fish’s distance from the edge of the dish was measured (Fig. 6*B1*). The mean position of the 20 larvae at 31.5 min after the start of AITC treatment (30-s period) was calculated (Fig. 6*B2*). The position of the fish after AITC treatment in the presence of IBU (IBU-AITC_THIGMO_ group = 7.02 ± 0.60 mm) was not significantly different from that of the group exposed to DMSO (DMSO-AITC_THIGMO_ group = 7.84 ± 0.52 mm; *p* > 0.05). Interestingly, IBU by itself appeared to enhance thigmotaxis in the larvae: the average position of the group treated with IBU, but not exposed to AITC (IBU-E3_THIGMO_ group = 8.22 ± 0.39 mm), was closer to the dish’s edge than that of the group treated with DMSO also without prior exposure to AITC (DMSO-E3_THIGMO_ group = 10.03 ± 0.64 mm; *p *<* *0.05; Fig. 6*B3*). Our data indicate that AITC-induced enhancement in thigmotaxis is unlikely to result from inflammatory processes; in addition, IBU alone appears to induce some AITC-independent enhancement of thigmotaxis in the larvae.

Finally, we tested the effect of IBU on the AITC-elicited increase in heart rate. Fish were initially exposed to 50 μm IBU or 0.1% DMSO for 30 min (Fig. 6*C1*). After this initial period of exposure to the compounds, fish were restrained in agarose as described above. Then the fish were given 30 min to acclimate to being restrained in agarose during which they remained in 50 μm IBU/0.1% DMSO. At the end of this period of acclimation, both groups were exposed to 10 μm AITC/E3 (Fig. 6*C2*). After 1 min, the AITC/E3 was washed out of the holding dish for 1 min with fresh E3 while the respective concentrations of IBU or DMSO were maintained. The heart rate of the larvae was measured during three 30-s periods that began 1 min before the start of exposure to AITC/E3, 30 s after the start of AITC/E3 exposure, and after washout procedures (4 min after the start of the exposure to AITC/E3). The normalized heart rate of fish during treatment with AITC in the presence of the anti-inflammatory drug (IBU-AITC_HR_ group) was significantly (*p *<* *0.05) enhanced (1.191 ± 0.013 BPM) compared with that of fish during treatment with AITC in the presence of DMSO (DMSO-AITC_HR_ group = 1.159 ± 0.009 BPM); moreover, this difference persisted for the 4-min test (AITC-IBU_HR_ group = 1.176 ± 0.010 BPM; DMSO-AITC_HR_ group = 1.137 ± 0.006 BPM; *p *<* *0.05). To assess the effect of IBU alone on heart rate, we treated fish with either IBU (50 μm) or DMSO (0.1%) without AITC exposure (Fig. 6*C3*). The heart rate of fish that received IBU alone was not significantly different from that of fish that received DMSO alone, either during the time of treatment (IBU-E3_HR_ group = 1.016 ± 0.009 BPM; DMSO-E3_HR_ group = 1.022 ± 0.006 BPM) or at the 4-min posttest after washout. Thus, by itself IBU had no effect on the heart rate of the larvae. In summary, IBU did not block the persistent increase in heart rate caused by AITC, producing, rather, a minor increase in the irritant-induced heart rate. From these data, we conclude that the apparent sensitization of heart rate in larvae that we observed following treatment with AITC cannot be explained by an inflammatory action of this chemical irritant.

A summary of the statistical analyses of the data presented in Figures 1-6 is presented in Table 7.

**Table 7:** Statistical analyses

	Data structure	Type of test	Power (α = 0.05)
a(Fig. 1*A2*)	Normally distributed	One-way ANOVA test	1.00
b(Fig. 1*A3*)	Normally distributed	One-way ANOVA test	0.98
c(Fig. 1*B2*)	Non-normally distributed	Two-way ANOVA test (Interaction)	0.63
d(Fig. 1*B2*)	Non-normally distributed	Two-way ANOVA test (main effect)	1.00
e(Fig. 1*B3*)	Non-normally distributed	Two-way ANOVA test (interaction)	0.14
f(Fig. 1*B3*)	Non-normally distributed	Two-way ANOVA test (main effect)	1.00
g(Fig. 1*C2*)	Non-normally distributed	Unpaired *t* test	0.60
h(Fig. 2*A2*)	Non-normally distributed	Unpaired *t* test	0.74
i(Fig. 2*A3*)	Non-normally distributed	Unpaired *t* test	0.77
j(Fig. 2*B2*)	Non-normally distributed	Unpaired *t* test	0.60
k(Fig. 2*C2*)	Normally distributed	Repeated-measures ANOVA test (interaction) normalized data	0.56
l(Fig. 2*C2*)	Normally distributed	Repeated-measures ANOVA test (main effect) normalized data	0.73
m(Fig. 2*C2*)	Normally distributed	Repeated-measures ANOVA test (interaction) raw data	0.79
n(Fig. 3*A*)	Normally distributed	Repeated-measures ANOVA test (interaction)	0.99
o(Fig. 3*B*)	Normally distributed	Unpaired *t* test	0.88
p(Fig. 4*A*)	Non-normally distributed	Repeated-measures ANOVA test (main effect)	1.00
q(Fig. 4*B*)	Non-normally distributed	Repeated-measures ANOVA test (interaction)	1.00
r(Fig. 5*A*)	Normally distributed	Repeated-measures ANOVA test (main effect)	1.00
s(Fig. 5*B*)	Normally distributed	Repeated-measures ANOVA test (main effect)	0.09
t(Fig. 6*A2*)	Normally distributed	Unpaired *t* test	0.07
u(Fig. 6*A3*)	Normally distributed	Unpaired *t* test	0.12
v(Fig. 6*B2*)	Normally distributed	Unpaired *t* test	0.17
w(Fig. 6*B3*)	Non-normally distributed	Unpaired *t* test	0.65
x(Fig. 6*C2*)	Normally distributed	Repeated-measures ANOVA test (main effect)	0.66
y(Fig. 6*C3*)	Normally distributed	Repeated-measures ANOVA test (main effect)	0.07

### Neural activity in a hindbrain region correlates with behavioral sensitization

**Figure 7.**
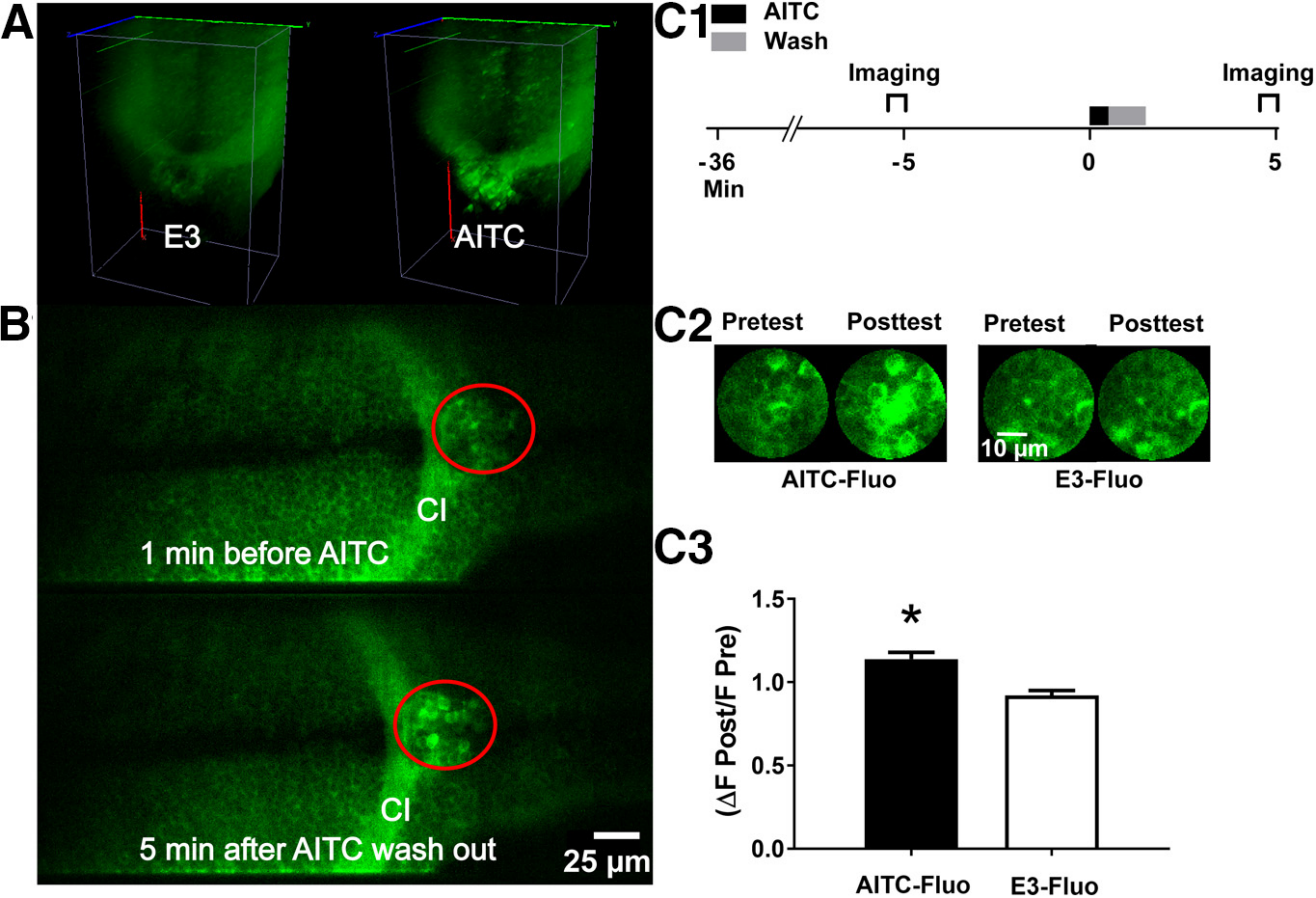
AITC causes an increase in neuronal activity that persists after washout in a hindbrain region of the larval brain. ***A***, Optical recordings of the hindbrain in a GCaMP6 transgenic larva made with a high-speed line scanning confocal fluorescence microscope. 3D reconstructions of a volume of the hindbrain (200 × 140 × 100 μm^3^) are shown in a larva before and during exposure to AITC. Images were recorded at 5 vols/s at 200 Hz. The images were collected at 1 min before (“E3”) during, and after AITC application. AITC induced strong activation of neurons throughout this brain region. ***B***, Sections from the volume recordings in ***A***. The region of interest (ROI; red circle) was just caudal to the commissura infima Halleri (CI; see Arrenberg et al., 2009). Scale bar, 25 μm. ***C1***, Protocol for examining potential sensitization-related activity in the ROI. Here, activity within the ROI shown in ***B*** was imaged for 1 min starting at 6 min before a 30-s exposure to AITC/E3 and at 2.5 min after washout (1 min long) of the AITC. ***C2***, Sample images taken of the ROI before (pretest) and after (posttest) exposure to E3 (images at left) or AITC (images at right). Scale bar, 10 μm. ***C3***, Persistent, postwashout effect of AITC on neuronal activity in the ROI. The normalized fluorescence was significantly greater following exposure to the chemical irritant (AITC-Fluo group, *n *=* *8) than following exposure to E3 (E3-Fluo, *n *=* *8; *t*_(14)_ = 4.80, *p *=* *0.0003). This figure shows means ± SEM; * indicates a significant (*p *<* *0.05) difference between groups.

To identify candidate neural circuits that might mediate AITC-induced behavioral sensitization, we used fish that express GCaMP6s under the *ELAV3* pan-neuronal promoter (Vladimirov et al., 2014) together with high-speed confocal microscopy. We imaged neural activity in the hindbrain and rostral portions of the spinal cord in GCaMP6s-expressing larvae (5 dpf) fully restrained in agarose before, during, and after exposure to AITC (10 μm). Based on these imaging experiments, we identified a small region at the border between the hindbrain and spinal cord that showed a strong change in fluorescence when the larval fish were exposed to AITC (Fig. 7*A*). Neural activity persisted in this region for ≥5 min after the washout of AITC (Fig. 7*B*); this persistent activity potentially represents a correlate of short-term behavioral sensitization. Interestingly, this region, which was located in the caudal-most part of the reticular formation, has previously been linked by Arrenberg et al. (2009) to the initiation of swimming, a behavior we found to be enhanced by AITC (Figs. 1, 2). To quantify the sensitization-related neural activity in this region, we treated larvae with AITC (10 μm, 30 s) and optically recorded (1.55-Hz sampling rate) from a small area (1075 μM^2^) just caudal to the commissura infima Halleri, which represents the border between the spinal cord and hindbrain (Fig. 7*C1*). The area from which we recorded contained ∼20 neurons (Fig. 7*C2*). We observed a significant increase in overall normalized fluorescence (ΔF/F) ∼3 min after AITC had been washed from the bath (AITC-Fluo group = 1.14 ± 0.04) compared with the change in fluorescence in this area in a control group exposed only to E3 (E3-Fluo group = 0.92 ± 0.03; Fig. 7*C3*). Persistent neural activity in this region may therefore mediate, at least in part, AITC-elicited sensitization of locomotion in zebrafish larvae.

## Discussion

In recent years, substantial progress has been made toward the localization of the engram, the physical memory trace (Semon, 1921; Lashley, 1929), for various types of learning and memory (Poo et al., 2016; Asok et al., 2019; Josselyn and Tonegawa, 2020). Nonetheless, we still lack a complete biological understanding of any form of memory, regardless of how simple, in any organism (Glanzman, 2009; Rankin et al., 2009). Given this situation, it is important to develop novel forms of learning in model systems, such as the larval zebrafish, amenable to the use of new cellular tools for investigations of the biological substrates of learning and memory (Aizenberg and Schuman, 2011; Roberts et al., 2011, 2013, 2016, 2019; Wolman et al., 2011, 2014, 2015; Ahrens et al., 2012; Marsden and Granato, 2015). Here, we have demonstrated, for the first time, a form of nonassociative learning in zebrafish larvae, sensitization (Groves et al., 1969) of locomotor behavior and heart rate. In addition, using transgenic fish that pan-neuronally express a calcium indicator, we identified a neural correlate of sensitization, specifically, a persistent increase in neuronal activity in a region of the zebrafish brain just caudal to the hindbrain-spinal cord border; this region was previously shown to initiate swimming in the larval zebrafish (Arrenberg et al., 2009). The persistent increase in activity induced by AITC in this region may represent a component of the engram for short-term sensitization in zebrafish larvae. It is also possible, however, that the postwashout enhancement of activity in this hindbrain region is merely downstream of another, more central, brain region where the memory for sensitization actually resides (see below).

Increased thigmotaxis in larval zebrafish can be triggered by stressful stimuli (Schnörr et al., 2012) or exposure to anxiogenic drugs (Richendrfer et al., 2012; Schnörr et al., 2012). Conversely, anxiolytic drugs, such as ethanol and Diazepam, decrease thigmotaxis (Richendrfer et al., 2012; Schnörr et al., 2012; Johnson and Hamilton, 2017). We observed that exposure to an aversive stimulus, the chemical irritant AITC, enhanced thigmotaxis in zebrafish larvae; this effect had an onset latency of 30 min. A possible explanation for the delay is that the onset of enhanced thigmotaxis was masked by AITC-induced sensitization of locomotion. Consistent with this idea, we observed that the AITC-elicited increase in locomotion was substantially reduced 30 min after removal of AITC from the bath (Fig. 2). In other words, an increase in the frequency of movement may have disrupted any thigmotactic tendency on the part of the fish, which only became apparent once locomotion returned to near baseline levels. The absence of significant thigmotaxis at times later than 30 min reflects the short-term nature of this memory.

The enhancement in heart rate produced by AITC (Fig. 4) indicates activation of the autonomic nervous system, which is functional early in zebrafish development (Mann et al., 2010). Although it is reasonable to attribute the persistence of increased heart rate in the larvae following washout of AITC to sensitization-related prolongation of autonomic nervous system activity, other interpretations of this effect are also possible. For example, given the lipid solubility of AITC, it is difficult to be certain that the chemical agent was entirely removed during washout of the AITC-containing solution. Previously, however, Prober et al. (2008) reported that RR can effectively block AITC-driven responses even if the RR is added to a bath already containing AITC. If residual AITC were mediating the increase in heart rate following the putative washout of the irritant in our experiments (Fig. 5*B*), one would have expected to observe a substantial reduction in normalized heart rate, which we did not. This result strongly argues against the idea that the prolonged enhancement of heart rate we observed was because of the residual presence of AITC in the bath. We believe that the sensitization-like effects of the irritant on locomotion and thigmotaxis in zebrafish larvae (Figs. 2–4) are similarly unlikely to have been because of residual AITC.

Because AITC can elicit inflammatory processes, such processes are potential causes of the behavioral changes elicited by AITC in the present study. The failure of the anti-inflammatory agent IBU to block any of the AITC-induced changes in behavior (Fig. 6), however, argues that the brief exposure to a relatively low dose of AITC used in our experiments either did not elicit substantial levels of inflammation in zebrafish larvae, or, if it did, that inflammation-related processes did not contribute significantly to the observed behavioral changes. However, IBU likely does not prevent isothiocyanate reactivity as isothiocyanates are highly reactive with sulfide groups (Hinman et al., 2006; Karlsson et al., 2016), leaving the possibility that non-IBU-sensitive inflammatory processes may have contributed to the behavioral results. Indeed, much longer exposures than used in the present study (hours-to-days) to AITC can deplete glutathione or other antioxidants (Overby et al., 2015). A reduction in antioxidants and other processes elicited by AITC exposure can have serious adverse effects on development, including malformations of the body in embryos (Williams et al., 2015).

Another possible explanation for the behavioral and physiological changes we observed following treatment with AITC is that they were because of minor injury caused by the chemical irritant. Evaluating this possibility is complicated by the fact that sensitization and the response of the nervous system to injury/irritation can involve similar, or even identical, neurobiological mechanisms. In *Aplysia*, where this issue has been examined in detail by Walters and colleagues, it has been shown that behavioral sensitization, because of electrical tail shocks that do not cause tissue damage, and frank damage of the nervous system, because of crush of the peripheral nerves that contain the axons of mechanosensory neurons, result in similar long-term cellular changes in mechanosensory neurons (Walters et al., 1991; Walters and Ambron, 1995); these changes include hyperexcitability, attributable to a decrease in the “S”-type potassium current (*I*_K,S_) in the sensory neurons (Ungless et al., 2002), and facilitation of transmitter release from the sensory presynaptic terminals. The available evidence indicates that the molecular pathways activated by injury and those activated by noxious stimuli that induce sensitization overlap to a significant extent. This has led to the speculation that some of the mechanisms underlying some simple forms of memory may have evolved from processes that originally subserved adaptation to neural injury (Walters et al., 1991). Nevertheless, we believe that the results of the tests of the effects of RR, as well as of IBU (Figs. 5, 6), on the alterations in behavior and heart rate in zebrafish larvae permit us to conclude that these changes resulted from learning rather than minor injury.

Behavioral sensitization in murine systems has been associated with the central nucleus of the amygdala, a forebrain structure (Koch and Ebert, 1993), as well as with the midbrain central gray (Fendt et al., 1994). A homolog of the mammalian amygdala has been identified in the pallium of the zebrafish (Perathoner et al., 2016), and there is experimental evidence linking this structure to regulation of thigmotaxis in larval zebrafish (Best et al., 2008). Furthermore, activity in the left dorsal habenulo-interpeduncular pathway attenuates fear-related freezing to an electrical shock in zebrafish larvae (Duboué et al., 2017). This suggests that central brain structures are likely to play roles in behavioral sensitization in the larval zebrafish, an idea that we plan to investigate in the future. It would be interesting to know in this regard whether the hindbrain region implicated in behavioral sensitization here (Fig. 7) is neurally connected to the pallium and/or the left habenula in the larval brain.

In addition, we will use cellular electrophysiological methods to determine how AITC increases the firing of neurons in the region shown in Figure 7. Possible mechanisms of locomotor sensitization in zebrafish, suggested by studies of the marine invertebrate *Aplysia*, include synaptic facilitation of the afferent input to, and enhanced excitability of, the excitatory neurons in the region (Byrne and Hawkins, 2015). In *Aplysia*, sensitization-related neuronal changes are mediated by the monoaminergic transmitter serotonin (Brunelli et al., 1976; Hochner et al., 1986a,b; Glanzman et al., 1989; Marinesco and Carew, 2002). In general accordance with this mechanistic scheme, we have recently found that dopamine receptors D4 and D1/D5 play critical roles in AITC-induced sensitization of locomotion in zebrafish larvae (our unpublished data).

In summary, we have discovered that a noxious stimulus, the irritant AITC, causes a form of behavioral sensitization in zebrafish larvae that is reflected in increases in locomotion, heart rate, and thigmotaxis, and that persists for ≤30 min. In addition, we have identified a specific neural correlate of this short-term learning: persistently increased activity in a brain region previously linked to locomotion in the larvae (Arrenberg et al., 2009). These results set the stage for a systems-level analysis of the formation and maintenance of a simple, nonassociative form of learning and memory in an experimentally tractable vertebrate model system.

## Synthesis

Reviewing Editor: Elizabeth Cropper, Icahn School of Medicine at Mt. Sinai Decisions are customarily a result of the Reviewing Editor and the peer reviewers coming together and discussing their recommendations until a consensus is reached. When revisions are invited, a fact-based synthesis statement explaining their decision and outlining what is needed to prepare a revision will be listed below. The following reviewer(s) agreed to reveal their identity: Thomas Abrams.

The reviewers felt most of the earlier comments have been adequately addressed. As listed below there are a few outstanding minor concerns. In particular please note the comments that were made during the Consultation session (which follow the comments of the individual reviewers).

Reviewer #1:

The modifications in this revised MS have substantially improved it. Several of the key arguments that the authors make are much more effective. The ibuprofen control is an important addition to discount the likelihood of a contribution from inflammation. The lack of effect of ruthenium red after AITC washout is a strong argument that washout was effective.

Most of the comments below are very minor. One more important comment concerns the statistical comparison of the time course of sensitization.

The presentation of statistics analysis of the time course of sensitization (l. 285-290) is not optimal. Presently, the conclusion is quite weak. “The interaction between exposure condition and time of testing was not significant ... locomotion in the AITC-treated group was enhanced compared to the E3-treated group for up to 60 min after washout of AITC/E3 ...” This statement could be correct even if the increase in activity persisted only at the earliest post-test time. “The lack of a significant interaction did not permit a more fine-grain temporal resolution of the duration of the effect of sensitization on locomotion” The lack of a significant time x treatment interaction would limit comparisons among individual time points, but not between treatment conditions at single time points. In their analysis, the authors actually omitted measurements at the pre-AITC-exposure time point, as all results had the pretest values subtracted from them - which explains the lack of an interaction with time. It would be advantageous to include posthoc comparisons at individual time points between treatment conditions - at least for the late time points. Another way to assess persistence of sensitization at the later time points would be to average the activity (raw or normalized distance moved) for the 45 and 60 min time points (activity does not decrease at these late times). These values could be compared between AITC and control, with a t test. Depending on the results of the t test, this would justify the conclusion that activity was enhanced for at least 45 min after AITC exposure ended.

l. 93. “We measured tail movements in response to an ejection of bath solution (100 μL) ...” This solution was presumably directed at the head, which should be more clearly described.

l. 140. “The IBU was dissolved in E3 containing 0.1% DMSO to a final concentration of 50 μM” This reads as if the solid ibuprofen was added to E3 with very dilute DMSO, and then dissolved. Was there not a stock of ibuprofen dissolved in DMSO first? If so, what was the stock concentration?

The rebuttal states “The first microscope, a custom-built, line scanning confocal microscope, had the capability to image a relatively large volume of the zebrafish brain; we used this microscope to initially identify the hindbrain region as a potentially mnemonic structure” “Initially, we focused on the hindbrain because previous studies showed this area was strongly activated by AITC” (l. 153.) “We imaged neural activity in the hindbrain ... “ (l. 551) The Methods and Results could make it clearer that initially a larger area of the brain was surveyed, and then the authors focused on a region where they found increased activity correlated with the AITC exposure.

l. 617. “The absence of significant thigmotaxis at times later than 30 min may reflect the short-term nature of this memory.” “May reflect” sounds speculative, whereas clearly the change in thigmotaxis was restricted to this single time point. This could be a more straightforward statement.

l. 650. Ungless et al did not record single K channels in Aplysia sensory neurons.

l. 672. “Likely mechanisms of locomotor sensitization in zebrafish, based on studies of the marine invertebrate Aplysia, are synaptic facilitation of the afferent input to, and enhanced excitability of, the excitatory neurons ...” This seems like too strong a statement, as plasticity in Aplysia sensory neurons is not a strong prediction of mechanisms of plasticity in zebrafish. s

It would be helpful to include sample size in tables with data.

Reviewer #2

The authors have argued extensively that the results of using ruthenium red and ibuprofen support the contention that inflammation is probably not present or affecting the results. I would have been happier if the authors had actually tried to measure some direct index of inflammation instead of hopefully relying on ibuprofen. Without that, the enthusiastic interpretations seem a bit based on faith. But I think the question is likely to be resolved at some future point and that the answer, whatever it is, will be of significant interest. I thus concur that this paper already contains significant results of interest to the community.

Comments from Consultation Session

With respect to the ibuprofen results both reviewers felt that it is important that material be added. In particular, it would be helpful if the authors explicitly noted that the dose used was twice the concentration used in the earlier paper (Ellis), therefore certainly should have suppressed COX enzymes that mediate inflammation. However, it was also noted that ibuprofen does not suppress isocyanate reactivity and the consensus was that this should be mentioned as a caveat, and because it provides interesting search directions for future research. Below are some specific references that may be useful for this discussion “isothiocyanates are highly reactive with sulfide groups, as on cysteine in peptides (https://doi.org/10.1038/srep21203) and glutathione. AITC effectively depletes cellular glutathione (doi: 10.3389/fpls.2015.00277. It is not surprising that it is a likely teratogen (https://doi.org/10.1016/j.toxrep.2014.12.005).”
