# Erratum: Roberts et al., “Induction of Short-Term Sensitization by an Aversive Chemical Stimulus in Zebrafish Larvae”

**DOI:** 10.1523/ENEURO.0514-21.2021

**Published:** 2022-01-13

In the article, “Induction of Short-Term Sensitization by an Aversive Chemical Stimulus in Zebrafish Larvae,” by Adam C. Roberts, Joseph B. Alzagatiti, Duy T. Ly, Julia M. Chornak, Yuqi Ma, Asif Razee, Gohar Zavradyan, Umair Khan, Julia Lewis, Aishwarya Natarajan, Alisher Baibussinov, Jasmine Emtage, Meghna Komaranchath, Jared Richards, Michelle Hoang, Jason Alipio, Emma Laurent, Amit Kumar, C. S. Campbell, Rebecca Stark, Javier Carmona, Anjum Hussain, Courtney Scaramella, Jenan Husain, Reed Buck, Ava Jafarpour, Miguel Garcia, Steve Mendoza, Gerardo Sandoval, Brandon Agundez, Amanda Fink, Emily Deutsch, Sarah C. Hernandez, Katsushi Arisaka, and David L. Glanzman, which published online on October 1, 2020, Felicia Osadi was inadvertently omitted from the author line. Felicia Osadi should be listed before Emily Deutsch in the author line. Her affiliation is the Department of Integrative Biology and Physiology, University of California, Los Angeles, and her contribution is performed research. The corrected author line appears below, and the online version has been updated.

Adam C. Roberts, Joseph B. Alzagatiti, Duy T. Ly, Julia M. Chornak, Yuqi Ma, Asif Razee, Gohar Zavradyan, Umair Khan, Julia Lewis, Aishwarya Natarajan, Alisher Baibussinov, Jasmine Emtage, Meghna Komaranchath, Jared Richards, Michelle Hoang, Jason Alipio, Emma Laurent, Amit Kumar, C. S. Campbell, Rebecca Stark, Javier Carmona, Anjum Hussain, Courtney Scaramella, Jenan Husain, Reed Buck, Ava Jafarpour, Miguel Garcia, Steve Mendoza, Gerardo Sandoval, Brandon Agundez, Amanda Fink, Felicia Osadi, Emily Deutsch, Sarah C. Hernandez, Katsushi Arisaka and David L. Glanzman

